# Could combined rapid diagnostic testing for malaria and c-reactive protein be helpful for the diagnosis and management of febrile illnesses in children under-5 years of age in rural Burkina Faso?

**DOI:** 10.1186/s12879-022-07638-2

**Published:** 2022-12-19

**Authors:** Massa dit Achille Bonko, Ibrahima Karama, Francois Kiemde, Palpouguini Lompo, Zakaria Garba, Sibidou Yougbaré, Petra F. Mens, Halidou Tinto, Marc Christian Tahita, Henk. D. F. H. Schallig

**Affiliations:** 1 Institut de Recherche en Sciences de la Santé- Direction Régionale du Centre-Ouest/Unité de Recherche Clinique de Nanoro, IRSS-DRCO /URCN), Nanoro, Burkina Faso; 2 Department of Medical Microbiology, Experimental Parasitology Unit, Amsterdam University Medical Centers, Academic Medical Center at the University of Amsterdam, Amsterdam, The Netherlands

**Keywords:** Bacterial infections, Malaria, mRDT-*Pf*HRP-2, C-reactive protein, Febrile children under 5, Antibiotics

## Abstract

**Background:**

Febrile illnesses are among the most important reasons for medical consultation in sub-Saharan Africa and are frequently treated with antimicrobials due to the unavailability of appropriate diagnostic tools. This practice leads to antimicrobial resistance, with increasing mortality and morbidity as result. One of the few accessible diagnostic tools available in low resource settings is malaria rapid diagnostic tests (mRDTs) which contributed to reducing the over-prescription of anti-malarials, but cannot guide antibiotic prescriptions. To circumvent this problem, we explored whether combined testing with mRDT and c-reactive protein (CRP) could improve the diagnosis of febrile illnesses and subsequent prescription of antibiotics.

**Methods:**

Clinical specimens (blood, stool and urine) collected from 396 febrile children (axillary temperature of ≥ 37.5 °C) were analyzed with rapid diagnostic tests (malaria and CRP) and microbiology culture to establish the possible cause of fever. Actual antimicrobial prescriptions given to the children were compared with those that could be given based on combined CRP-malaria testing.

**Results:**

In total, 68.7% (272/396) of malaria cases were diagnosed by mRDT-*Pf-*HRP-2. CRP test was positive in 84.3% (334/396) of the children, but bacterial infections were confirmed in 12.4% (49/396) of them. A possible cause of fever could not be established in 20.5% (81/396) of cases. Based on the diagnostic practice in place, 265 of the children with a positive mRDT-*Pf-*HRP-2 received anti-malarial treatment. Furthermore, 89.5% (111/124) of negative mRDT results received antibiotic treatment and 37.1% (46/124) received antimalarial treatment. Of these 124 cases, 80 had positive CRP tests and 44 negative CRP tests. If the results of CRP testing are considered, 44 CRP/mRDT negative children would not get antibiotic treatment, resulting in a 35.5% reduction in antibiotic prescriptions. However, 2 cases with a bacterial infection would be denied appropriate treatment.

**Conclusion:**

Combining mRDT-*Pf*HRP2 with CRP testing is particularly useful in children for whom both tests are negative as it results in a reduction of antibiotics prescriptions. However, there is a risk to miss potential severe bacterial infections and a close follow-up of these cases is strongly recommended.

## Background

In Burkina Faso, like in many other sub-Sahara African countries, febrile illnesses in children are amongst the foremost reasons for medical consultation [1–3]. In resource limited settings, with inadequate availability of appropriate diagnostic tools for a proper diagnosis of the actual cause of fever, antimicrobials are often systematically prescribed. This unwanted use of antimicrobials results in increasing resistance with as main consequence thousands of deaths caused by resistant pathogens in Africa and Asia [4, 5].

In Nanoro, a rural malaria-endemic area, many cases of fever have been reported in children under 5 years of age with many of them being malaria infection [1, 6]. Next to malaria, bacterial, viral, or other parasitic infections are also common and may cause fever [7, 8]. However, only bacterial febrile infections should be treated with antibiotics, as for other febrile infections the use of antibiotics may not be justified [5, 9]. In such context, proper diagnosis is therefore essential in order to make right treatment decisions. Nevertheless, in many rural and resource limited settings apart from the malaria rapid diagnostics tests (RDTs) [6, 9–12], there are not many diagnostic tools available to support the clinical diagnosis of other febrile diseases. Consequently, antibiotics are regularly prescribed to malaria-negative cases without knowing the actual cause of fever and this contributes to increasing antibiotic resistance.

In order to tackle antimicrobial resistance, there is a pressing need to develop better diagnostic strategies to improve the management of non-malaria fever in remote areas lacking suitable laboratory infrastructure. Next to malaria, it is highly relevant to develop diagnostic strategies for bacterial and viral infections as the later do not require treatment with antibiotics. C-reactive protein (CRP) is a relatively accurate and sensitive marker for inflammation and infection, and could be an indication of a (possible) bacterial infection [13–15]. The use of a RDT that can semi-quantitatively determine CRP levels could therefore support the diagnosis and management of febrile diseases and may reduce the inappropriate prescription and use of antimicrobials [16–20]. In bacterial infections with severe inflammations, CRP level is between 40 and 200 mg/L. Furthermore, in some severe bacterial infections or burns, CRP level can increase above 200 mg/L, after 48 h of the occurrence of an acute event [19]. In contrast, CRP level is in general between 10 and 40 mg/L in viral infections with mild inflammations [13, 14, 20].

In the routine practice in Burkina Faso rural health centers, febrile children are screened with a malaria RDT based on the detection of *Plasmodium falciparum* specific histidine-rich protein 2 (mRDT-*Pf*HRP2). RDT-positive cases are treated with appropriate anti-malaria drugs as recommended by the National Malaria Control Program (NMCP) guidelines [21]. Despite its relatively good sensitivity [6, 9–12], the use of *Pf*HRP2 RDT alone does not allow for the diagnosis of all causes of fever. Consequently, febrile cases tested negative for malaria by RDT are often systematically treated with antibiotics, which contributes to overuse of antimicrobials [22–25]. Moreover, local health workers sometimes also tend to prescribe antibiotics to mRDT-*Pf*HRP2-positive children in addition to antimalarials. This is might be due to the fact that the health workers want to be certain that they have adequately treated the febrile cases and that a child is not sent away with no or wrong medication [26]. This attitude contributes further to the over prescription of antibiotics. To circumvent this problem, we explored in this study whether a combination of screening with *Pf*HRP2 RDT and a semi-quantitative CRP RDT could improve the diagnosis of febrile illnesses in a population of feverish children under 5 years of age in a rural area in Burkina Faso and increases the adequate prescription of antimicrobials.

## Methods

### Study design

We prospectively included 396 children under 5 years of age with an axillary temperature of ≥ 37.5 °C in this study. Recruitment was carried out from April to December 2016 in the Nanoro Health District catchment area (Burkina Faso). Nanoro is a malaria-endemic area, located in the Central–West region of Burkina Faso at approximately 100 km from Ouagadougou, the capital city. This study was conducted in the framework of a large project (RAPDIF: a rapid diagnostic test for undifferentiated fevers; which is supported by a grant from the Netherlands Organisation for Health Research and Development (ZonMw), project 205300005), which aims to improve the diagnosis and management of febrile illness in children under 5 years of age [8].

A whole blood sample was collected from each included child for malaria screening (at enrolment) and CRP testing with RDTs, for blood culture and for malaria microscopy. In total 396 blood samples were collected and analyzed in this manner. In addition, we collected 226 stool samples and 224 urine samples from these children for microbiological analyses. All laboratory analyses were performed according to established standard operating procedures (SOPs) of the Clinical Research Unit of Nanoro (CRUN) laboratory. A primary clinical diagnosis for the management of febrile children was done by health facility nurses following the Integrated Management of Childhood Illness (IMCI) guidelines [26]. The experimental procedures (microbiological analysis, CRP testing and microscopy) were not taken into account when treatment was installed at the primary health care facilities. The prescription of antimicrobials (i.e., either an anti-malarial as recommended by the Burkinabe NMCP, or antibiotics, or both) was recorded on case record form (CRF). Whenever results of laboratory testing became available, these were communicated to the health facilities staff to allow them to adjust treatments (if needed) and to ensure better case management.

Written informed consent was obtained from a parent or legal guardian of each child before enrollment. The study protocol was approved by the National Ethical Committee for Health Research of Burkina Faso (Deliberation No. 2014-11-130).

### Laboratory procedures

To determine whether or not CRP testing added to malaria RDT testing could be helpful in defining the actual cause of febrile illness, we combined the result of the RDT-*Pf*HRP2 RDT and that of semi-quantitative CRP test (results categorized in three groups: < 10 mg/L, 10–40 mg/L, and > 40 mg/L) with those of bacterial bloodstream infections, bacterial gastroenteritis, bacterial urinary tract infections, parasitic gastroenteritis, and viral gastroenteritis.

Infections caused by *Salmonella species* isolated from blood and stool samples, *Escherichia coli* from blood and urine, *Streptococcus pneumoniae*, *Haemophilus influenzae* b, and *Enterobacter agglomerans* from blood are considered as infections that require treatment with antibiotics. Gastroenteritis caused by enteropathogenic *Escherichia coli* (EPEC) is not treated with antibiotics in Burkina Faso like in the majority of West African Countries [27, 28].

#### Malaria rapid diagnostic test

Malaria diagnosis was performed at enrolment with a *Pf*HRP2 protein based RDT (SD Ag Bioline *Pf*; Standard Diagnostics, Hagal-Dong, Korea) according to the manufacturer’s instruction. This RDT is recommended by the NMCP of Burkina Faso [21]. The results of mRDT-*Pf*HRP2 were (for the purpose of the present study) used for the definition of a malaria case as this is the routine diagnostic practice in the participating health centers. Test results were recorded on study CRFs.

#### Microscopy

Thin and thick blood smears were prepared from blood collected in Ethylene Diamine Tetra Acetic acid (EDTA) tubes and fixed with ethanol (thin slides) and stained with 3% Giemsa solution (pH 7.2). Two certified expert microscopists independently read the blood smears in order to search for *Plasmodium species*. In case of discordance, the slides were read by a third independent expert microscopist whose conclusion was decisive.

#### C-reactive protein (CRP) rapid test

CRP testing was performed using the semi-quantitative Actim® CRP RDT (Oy Medix Biochemical, Joensuu, Finland). This RDT allows for the semi-quantitative determination of CRP serum concentrations into 4 categories: (i) < 10 mg/L; (ii) between 10 and 40 mg/L; (iii) between 40 and 80 mg/L; (iv) and > 80 mg/L. Tests were performed according to the manufacturer’s instructions and read by two technicians who independently recorded the test results. The Actim® CRP RDT has an internal control line, which confirms the performance (accuracy) of the test.

#### Microbiology procedures

The microbiology procedures used for this study have been described in detail elsewhere [8]. Briefly, blood cultures were performed on 1 to 3 mL of venous blood samples collected in Pediatric blood culture bottles (BD BACTEC Peds PlusTM/F, Becton Dickinson, and Company, Sparks, Maryland, USA) from the enrolled children and incubated in a BACTEC 9050 instrument (Becton Dickinson) for 5 days according to the manufacturer’s instructions. Pathogens present in positive blood cultures were identified by standard microbiological and biochemical methods [29–31].

Fresh stool samples collected in sterile containers by study nurses were subject to culture, microscopy, and Rota/adenovirus RDT testing. Firstly, we cultured the stool on Sodium Selenite broth and on appropriate agar followed by an incubation at 35–37 °C for 18–24 h. Subsequently, we identified suspected colonies (i.e., *Salmonella* species, *Shigella* species*,* and enteropathogenic *Escherichia coli* [EPEC; only for children under 2 years]) by standard microbiological and biochemical methods (API system, bioMerieux Marcy-L’Etoile, France) [29–31]. Secondly, we performed a specific analysis of stools for the qualitative detection of group A rotavirus and adenovirus serotypes 40 and 41 in human stool samples using a Rota/Adenovirus antigen rapid diagnostic test (SD Bioline Rota/Adeno; Standard Diagnostic, Inc., Korea). Finally, we performed a microscopic examination of the stool to search for intestinal parasites.

Urine samples were collected in sterile containers and tested using urine dipsticks (Standard Diagnostics, Urocolor, Inc, Korea). All samples tested positive for leukocytes and nitrite were cultured on appropriate agar and incubated for 18–24 h at 35–37 °C. Samples that resulted pure bacterial growth of > 10^5^ colonies forming units (CFU)/mL were considered significant bacteriuria [32] and we identified the bacterial isolates according to biochemical methods (API system, bioMerieux Marcy-L’Etoile, France).

### Quality control procedures

To ensure the quality of the samples’ analysis, all experiments were conducted according to the standard laboratory operating procedures (SOPs) in place at CRUN laboratory. Furthermore, there was strict adherence to manufacturers’ instructions. For the semi-quantitative CRP rapid test and mRDT-*Pf*HRP2, the internal control lines confirmed the correct performance of these tests. In addition, we perform monthly internal quality controls of reagents and equipment using American Type Culture Collection (ATCC) standard reference strains. It is important to mention that the CRUN laboratory is subjected to external quality control organized by world health organization (WHO) and national institute for communicable diseases (NICD, South Africa). Finally, at CRUN laboratory, microscopists are regularly submitted to an external quality control program on quarterly basis and only certified microscopists are allowed to perform malaria slide reading [33].

### Data analysis

Data were double entered from study CRFs into OpenClinica software. The data were subsequently analyzed using STATA® statistical software version13 StataCorp LLC, College Station, TX, USA. Qualitative variables description was performed using the percentages. The median and mean were used for quantitative variables distribution. The baseline parasite density was described using geometric means.

## Results

### Characteristics of study participants

The characteristics of study participants are summarized in Table 1, which also presents the results for diagnostic testing for CRP and malaria by RDT or expert microscopy.

**Table 1:** Basic characteristics of study participants

Characteristics	n (%)
Age (in months), median (IQR 25–IQR 75)	23 (12–36)
Male	223 (56.3)
Female	173 (43.7)
Weight (in kilograms), mean (SD)	9.87 (3.1)
Temperature, (N = 396), n (%)	
[37.5 °C–38.5 °C]	194 (49.0)
[38.5 °C–39.5 °C]	143 (36.1)
> 39.5 °C	59 (14.9)
Biological samples collected	
Blood sample	396 (100)
Stool sample	226 (70.1)
Urine sample	224 (56.6)
Malaria microscopy	
Positive	237 (59.9)
*Plasmodium falciparum* density, geometric mean (95%CI) μl^−1^	22,276 (16,788–29,557)
Negative	159 (40.1)
RDT-*Pf*HRP2	
Positive	272 (68.7)
Negative	124 (31.3)
CRP	
Negative	
Level < 10 mg/L	62 (15.7)
Positive	334 (84.3)
Level 10–40 mg/L	65 (19.5)
Level > 40 mg/L	269 (80.5)
Bacterial infections	49 (12.4)
Blood stream infection	23 (5.8)
Bacterial gastroenteritis	25 (11.1)
Urinary tract infection	1 (0.3)
Non-bacterial infections	74 (18.7)
Parasitic gastroenteritis	70 (31.0)
Viral gastroenteritis	4 (1.8)
Children with unknown cause of fever^a^	81 (20.5)

CRP: C-reactive protein; RDT-PfHRP2: rapid diagnostic test for Plasmodium falciparum-specific histidine-rich protein 2; IQR: interquartile range; SD: standard deviation; CI: confidence interval

^a^Children having negative malaria testing (RDT-PfHRP2 and microscopy) and negative microbiology analyses (Bacterial infections and Non-bacterial infections)

Results of microbiology laboratory analyses are also presented in Table 1 It was found that 64.0% (16/25) bGEs were caused by EPEC not requiring antibiotic treatment. However, 67.3% (33/49) of the bacterial infections must be treated with antibiotics (Table 2). Furthermore, non-bacterial infections (i.e., either a parasitic gastro-enteritis [pGE] or a viral gastro-enteritis [vGE]; see Table 2) were confirmed in 18.7% (74/396) of febrile children. Finally, there were 81 febrile cases (20.5%; 81/396) of whom a possible cause of febrile illness could not be established.

**Table 2:** Distribution of laboratory results according to CRP levels

Laboratory findings	Negative CRP level	Positive CRP level	
	< 10 mg/L	10–40 mg/L	> 40 mg/L
Malaria RDT *Pf*HRP2 (N = 396), n (%)	62 (15.7)	65 (16.4)	269 (67.9)
Positive (n = 272), n (%)	18 (6.6)	33 (12.1)	221 (81.3)
Negative (n = 124), n (%)	44 (35.5)	32 (25.8)	48 (38.7)
Bacterial infections (n = 49), n (%)	7 (14.3)	6 (12.2)	36 (73.5)
bBSI (n = 23), n (%)	1 (4.3)	3 (13.1)	19 (82.6)
bGE (n = 25), n (%)	5 (20)	3 (12.0)	17 (68.0)
bUTI (n = 1), n (%)	1 (100)	0 (0.0)	0 (0.0)
Non-bacterial infections (n = 74), n (%)	11 (14.9)	14 (18.9)	49 (66.2)
pGE (n = 70), n (%)	10 (14.3)	13 (18.6)	47 (67.1)
vGE (n = 4), n (%)	1 (25.0)	1 (25.0)	2 (50.0)
Children with one or more infection(s) (n = 311), n (%)	30 (9.6)	41 (13.2)	240 (77.2)
Malaria alone (as determined by RDT) (n = 196), n (%)	14 (7.1)	22 (11.2)	160 (81.6)
pGEs alone (n = 16), n (%)	6 (37.5)	3 (18.75)	7 (43.75)
bBSIs alone (n = 11), n (%)	1 (9.1)	3 (27.3)	7 (63.6)
bGEs alone (n = 6), n (%)	3 (50.0)	1 (16.7)	2 (33.3)
vGEs alone (n = 3), n (%)	0 (0.0)	1 (33.3)	2 (66.7)
Malaria combined with either a bacterial or a parasitic infection (n = 76), n (%)	4 (5.3)	11 (14.7)	61 (80.3)
No malaria infection but either a bacterial infection combined with a parasitic infection (n = 2) or a combination of a viral infection with a bacterial infection (n = 1), n (%)	2 (66.7)	0 (0.0)	1 (33.3)
Children with unknown cause of fever^a^ (n = 81), n (%)	30 (37.0)	23 (28.4)	28 (34.6)

CRP: C-reactive protein; RDT-PfHRP2: rapid diagnostic test for *Plasmodium falciparum*-specific histidine-rich protein 2; bBSI: bacterial bloodstream infection; bGE: bacterial gastroenteritis; bUTI: bacterial urinary tract infection; pGE: parasitic gastroenteritis; vGE: viral gastroenteritis

^a^Children having negative malaria testing (RDT-PfHRP2 and microscopy) and negative microbiology analyses (Bacterial infections and non-bacterial infections)

### Laboratory results categorized according to CRP levels

Most of the children who were tested RDT positive for malaria also had a CRP positive test (93.4%; 254/272). But of those who were tested negative for malaria by RDT, 35.5% (44/124) also had a negative CRP test (see Table 2). In contrast, 80 (64.5%) cases that were mRDT negative had a positive CRP test. Furthermore, the majority of children for whom a bacterial infection was confirmed by microbiology analyses, were also tested positive with the CRP test (85.7%; 42/49). In the case of confirmed non-bacterial infections, positive CRP levels were found in 85.1% (63/74) of the febrile children. Altogether, a relatively low number of febrile cases with confirmed infection, either a bacterial infection (7/49; 14.3%) or a non-bacterial (11/74; 14.9%) were tested negative for CRP (< 10 mg/L).

In febrile children where no infection was diagnosed (20.5%; 81/396), and whose actual cause of fever remained unknown, the CRP test was positive in 63.0% (51/81) of the cases and negative in 37.0% (30/81) of the children (Table 2).

### Distribution of mRDT-PfHRP2 and RDT-CRP results over non-malaria infections

A more detailed distribution over the number of positive/negative mRDT-*Pf*HRP2 tests and CRP testing over the different types of non-malaria infections found by microbiology analysis is presented in Table 3. In febrile children with a positive mRDT-*Pf*HRP2 test (68.7%; 272/396), we also diagnosed a total of 30 bacterial infections but all are not treated with antibiotics. Among those, 12 bBSI and 18 bGE were diagnosed of which 10 bGEs were caused by EPEC that is not treated in accordance with the Burkinabe treatment guidelines. Furthermore, we also diagnosed 52 non-bacterial infections in mRDT-*Pf*HRP2 positive cases.

**Table 3:** Distribution of mRDT-*Pf*HRP-2 and semi-quantitative RDT-CRP tests results over non-malaria infections

Laboratory result (Infection type)	mRDT *Pf*HRP2 (N = 396)		Negative mRDT–*Pf*HRP2 and positive CRP test; (N = 80)		Negative mRDT–*Pf*HRP2 and negative CRP test; (N = 44)
	Positive (n = 272)	Negative (124)	CRP level 10–40 mg/L	CRP level > 40 mg/L	
BIs (n = 49)	30 (61.2)	19 (38.8)	4 (8.2)	10 (20.4)	5 (10.2)
bBSI (n = 23), n (%)	12 (52.2)	11 (47.8)	3 (13.0)	7 (30.4)	1 (4.3)
*NTS* (n = 15)	9 (60)	6 (40)	2 (13.3)	3 (20.0)	1 (6.7)
*TS* (n = 2)	1 (50.0)	1 (50.0)	0 (0.0)	1 (50.0)	0 (0.0)
*E. coli* (n = 3)	1 (33.3)	2 (66.7)	1 (33.3)	1(33.3)	0 (0.0)
*H. influenza* (n = 1)	0 (0.0)	1 (100.0)	0 (0.0)	1 (100.0)	0 (0.0)
*E. agglomerans* (n = 1)	0 (0.0)	1 (100.0)	0 (0.0)	1 (100.0)	0 (0.0)
*S. pneumonia* (n = 1)	1 (100.0)	0 (0.0)	0 (0.0)	0 (0.0)	0 (0.0)
bGE (n = 25), n (%)	18 (72.0)	7 (28.0)	1 (4.0)	3 (12.0)	3 (12.0)
*NTS* (n = 9)	8 (88.9)	1 (11.1)	1 (11.1)	0(0.0)	0 (0.0)
*EPEC* (n = 16)	10 (62.5)	6 (37.5)	0 (0.0)	3 (18.75)	3 (18.75)
bUTI (n = 1), n (%)	0 (0.0)	1 (100.0)	0 (0.0)	0 (0.0)	1 (100.0)
*E. coli* (n = 1)	0 (0.0)	1 (100.0)	0 (0.0)	0 (0.0)	1 (100.0)
nBIs (n = 74), n (%)	52 (70.3)	22 (29.7)	4 (5.4)	10 (13.5)	8 (10.8)
pGE (n = 70), n (%)	52 (74.3)	18 (25.7)	3 (4.3)	8 (11.4)	7 (10.0)
*Giardia intestinalis* (n = 32)	23 (71.9)	9 (28.1)	1 (3.1)	5 (15.6)	3 (9.4)
*Entamoeba species* (n = 20)	14 (70.0)	6 (30.0)	2 (10.0)	2 (10.0)	2 (10.0)
*Trichomonas intestinalis* (n = 13*)*	11 (84.6)	2 (15.4)	0 (0.0)	1 (7.7)	1 (7.7)
*Endolimax nana* (n = 4)	3 (75.0)	1 (25.0)	0 (0)	0 (0)	1 (25.0)
*Ascaris *sp*.* (n = 1)	1 (100.0)	0 (0)	0 (0)	0 (0)	0 (0)
vGE (4), n (%)	0 (0.0)	4 (100.0)	1 (25.0)	2(50.0)	1 (25.0)
*Adenovirus species* (n = 4)	0 (0.0)	4 (100.0)	1 (25.0)	2(50.0)	1 (25.0)
Unknown causes of fever^a^ (n = 81), n (%)	0 (0.0)	81 (100)	23 (28.4)	28 (34.6)	30 (37.0)

CRP: C-reactive protein; mRDT-PfHRP2:malaria rapid diagnostic test for *Plasmodium falciparum*-specific histidine-richprotein 2; BIs: bacterial infections; nBIs; non bacterial infections; bBSI: bacterial bloodstream infection; bGE: bacterial gastroenteritis; bUTI: bacterial urinary tract infection; pGE: parasitic gastroenteritis; vGE: viral gastroenteritis;: NTS: non-thypoid salmonella; TS: typhoid salmonella; EPEC: enteropathogenic *Escherichia coli*

^a^Children who have no infections (bacterial, non-bacterial, and malaria) diagnosed

In febrile children with a confirmed bacterial infection and who were tested negative with mRDT-*Pf*HRP2 (n = 19), there were 14 cases who were tested CRP positive and 5 were tested CRP negative. There were 22 cases of confirmed non-bacterial infection of whom 14 were tested mRDT-*Pf*HRP2 negative but CRP positive and 8 who were tested negative for both RDTs.

Of the 81 cases who were tested negative with mRDT-*Pf*HRP2 and for whom the cause of fever could not be explained by the identification of an infective agent, there were 51 cases (63.0%) with a positive CRP test and 30 cases (37.0%) with a negative CRP test.

### Prescription of antimicrobials by health staff

All cases (n = 272; see Table 4) with a positive mRDT-*Pf*HRP2 should have been treated with anti-malarials. Indeed, 265 (97.4%) of the children in this group did actual receive anti-malarials, but 7 children (2.6%) did not but the reason for this was not recorded in the CRFs (see Table 4). Furthermore, there were 147 (54.0%) malaria cases who were also additionally treated with one antibiotic and 10 (3.7%) with even 2 additional antibiotics (see Table 4).

**Table 4:** Antimicrobial prescriptions done by health workers for the febrile children

Number of. antimicrobial(s) prescribed	General antibiotic prescriptions in febrile children, (N = 269)							
	mRDT-*Pf*HRP 2, (N = 396)		Bacterial infections, (N = 49)			CRP results, (N = 396)		
	Positive (n = 272), n (%)	Negative (n = 124), n (%)	bBSIs (n = 23), n (%)	bGEs (n = 25), n (%)	bUTIs, (n = 1), n (%)	Level < 10 mg/L (n = 62), n (%)	10-40 mg/L (n = 65), n (%)	Level ≥ 40 mg/L (n = 269), n (%)
No antimalarial prescribed, (N = 85)	7 (2.6)	78 (62.9)	8 (34.8)	6 (24.0)	1 (100.0)	29 (46.8)	19 (29.2)	37 (13.8)
Antimalarial prescribed, (N = 311)	265 (97.4)	46 (37.1)	15 (65.2)	19 (76.0)	0 (0.0)	33 (53.2)	46 (70.8)	232 (86.2)
No antibiotic prescribed, (n = 128)	115 (42.3)	13 (10.5)	7 (30.4)	11 (44.0)	0 (0.0)	13 (21.0)	15 (23.1)	100 (37.2)
Antibiotic prescribed, (N = 268)								
One antibiotic prescribed, (n = 235)	147 (54.0)	88 (71.0)	13 (56.5)	12 (48.0)	0 (0.0)	44 (71.0)	41 (63.1)	150 (55.8)
Two antibiotics prescribed (n = 32)	10 (3.7)	22 (17.7)	3 (13.0)	2 (8.0)	1 (100.0)	5 (8.1)	9 (13.8)	18 (6.7)
Three antibiotics prescribed (n = 1)	0 (0.0)	1 (0.8)	0 (0.0)	0 (0.0)	0 (0.0)	0 (0.0)	0 (0.0)	1 (0.4)

CRP***:**** C-*reactive protein; mRDT-PfHRP2: malaria rapid diagnostic test for *Plasmodium falciparum*-specific histidine-rich protein 2; bBSI: bacterial bloodstream infection; bGE: bacterial gastroenteritis; bUTI: bacterial urinary tract infection; pGE: parasitic gastroenteritis; vGE: viral gastroenteritis

Children who were tested negative with mRDT-*Pf*HRP2 (n = 124) should not get anti-malarial treatment, but there were 46 children (37.1%; 46/124) who still received anti-malarials. In addition, 88 (71.0%; 88/124) mRDT-*Pf*HRP2 negative children were treated with 1 antibiotic, 22 children (17.7%) in this group were treated with 2 antibiotics and 1 child even received treatment with 3 antibiotics, irrespective of knowing the actual cause of fever.

Based on the microbiology analyses we can also categorize what treatments were provided to the febrile children who actually had a bacterial infection. In the group of children with a confirmed bBSI (n = 23), there were 15 (65.2%) who received anti-malarials and 8 (34.8%) who did not. Importantly, 7 children (30.4%) with a confirmed bBSI did not receive antibiotic treatment, whereas 13 (56.5%) received 1 antibiotic and 3 (13.0%) were prescribed 2 antibiotics.

Nineteen children (76.0%; 19/25) with a confirmed bGE received anti-malarials. In terms of antibiotics prescriptions, there were 11 children (44.0%) with a bGE who did not receive antibiotics, 12 (48.0%) were prescribed one antibiotic and two children (8.0%) with bGE received two antibiotics.

One child with a bacterial urinary tract infection did not receive antimalarials, but did receive two antibiotics.

### Effect of additional CRP testing on the prescription of antimicrobials

As a result of the customary diagnostic approach to manage febrile cases in place in the rural health centers in our study area, febrile children with a positive mRDT-*Pf*HRP2 are treated with anti-malarials and those with a negative malaria RDT with antibiotics. Consequently, we diagnosed 272 malaria cases by mRDT, who should receive anti-malarial treatment as recommended by the NMCP guidelines of Burkina Faso. Of note is that within this positive mRDT-*Pf*HRP2 group, 20 cases (7.4%) had also bacterial infection confirmed by microbiology analyses and that needed antibiotic treatment (see Table 5). However, due to the normal diagnostic approach in place, they have not been treated as such. In this respect, additional CRP testing will not improve the management of these particular 20 cases as per default they will only receive anti-malarial treatment on the basis of their positive mRDT result.

**Table 5:** Antibiotic prescriptions to febrile children with positive mRDT-*Pf*HRP-2 and confirmed bacterial infections

Number of antibiotic(s) prescribed	Number of children with positive mRDT-*Pf*HRP and bacterial infections and who received antibiotic prescriptions, (N = 20)^a^				
	Bacterial infections, (n = 20)		CRP results, (n = 20)		
	bBSI, (n = 12)	bGE, (n = 08)	Level < 10 mg/L, (n = 1)	10-40 mg/L, (n = 0)	Level ≥ 40 mg/L, (n = 19)
One antibiotic prescribed, (n = 9)	5 (41.7)	4 (50.0)	0 (0.0)	0 (0.0)	9 (47.4)
Two antibiotics prescribed, (n = 1)	1 (8.3)	0 (0.0)	0 (0.0)	0 (0.0)	1 (5.3)

CRP: C-reactive protein; mRDT-PfHRP2: malaria rapid diagnostic test for *Plasmodium falciparum*-specific histidine-rich protein 2; bBSI: bacterial bloodstream infection; bGE: bacterial gastroenteritis

^a^ Bacterial infections without bacterial gastroenteritis caused by enteropathogenic *Escherichia coli* (n = 10) (this infection is under the Burkinabe guidelines not treated)

Within the group of 124 children with a negative mRDT (and who were given antibiotics for relief), there were 64.5% (80/124) had a positive CRP test and 35.5% (44/124) had a negative CRP test (see Table 3). Therefore, if the results of additional CRP testing were considered for the prescription of antibiotics, the 44 children with a negative CRP test would not have received antibiotic treatment. As a result, this would lead to a reduction of 35.5% of antibiotic prescriptions in the group of febrile children with a negative mRDT-*Pf*HRP2. However, within this group, there were 2 febrile cases (a child with a non-typhoid salmonella blood infection and one with a urinary tract infection caused by *E. coli)*, who actually needed antibiotic treatment, but would on the basis of the negative result of the semi-quantitative CRP test not receive it.

## Discussion

Our study aimed to assess whether the combination of semi-quantitative RDT-CRP testing with mRDT-*Pf*HRP2 could improve the diagnosis of febrile illness in rural Burkina Faso, and consequently would lead to a more correct prescription of antimicrobials. Our present study indicates that this approach has proven to be of limited value in our study area. Most cases with malaria infection, also had a high CRP level, which confirms the results of other studies [14, 34, 35]. This makes difficult to detect an additional bacterial infection needing antibiotic treatment in child with a positive mRDT, particularly in our study area where the prevalence of malaria is very high. On the other hand, if the mRDT is negative and the CRP test is positive, malaria infection can indeed be ruled out in most cases. However, in such a case the prescription of antibiotics is not always warranted. For example, parasitic gastro-enteritis, for which antibiotic treatment is not indicated, can often increase the CRP levels [36]. Therefore, in the event of a suspected parasitic gastro-enteritis, microscopic examination of stool is highly recommended to better determine the actual cause of fever. In addition to this, microscopic testing for *Plasmodium* infection, the gold standard for malaria diagnosis, should be reconsidered in areas with high malaria prevalence and with the risk of significant numbers of false-positive mRDTs, in particular due to persisting HRP2 antigen [6, 11, 36]. Another potential drawback that threatens the use of HRP2-based mRDTs are potential false negatives results due to HRP2/HRP3 genes deletion or mutation [37]. However, according to a very recent systematic review and meta-analysis these mutations have not (yet) been found in Burkina Faso, but their potential occurrence needs to be carefully monitored [38].

The strength of combined mRDT and CRP testing is highlighted in our study in case both tests are negative. This was the case in 44 children of whom the majority of the children, did indeed not need either anti-malarial and/or antibiotics treatment. However, if this diagnostic approach is followed, two children will be denied essential antimicrobial treatment. In this respect, health policymakers, will have to consider whether not treating this particular small group of children outweighs the savings in prescribing antimicrobials in general or not.

In our study area, as was also previously reported malaria remains one of the most important causes of febrile illness [1, 39]. However, in this study, next to malaria, a significant proportion of febrile children were infected with bacterial pathogens or parasitic or viral gastroenteritis. There was also a group of children for whom the actual cause of febrile illness could not be established. A part of these children could have a viral infection responsible for fever, e.g., viral infection of the respiratory tract [39]. Unfortunately, we were not able to confirm these infections as our laboratory, like in many low-and middle-income countries, lacks the facilities to establish this, and this is a limitation of our study.

Another limitation of our work is that the current study design did not allow for statistical testing of sensitivity and specificity of the combined malaria and CRP testing. This is due to the fact that a majority of those recruited suffered from malaria and therefore will have inflammation ongoing. Perhaps performing a similar survey of the RDTs in a less malaria endemic region could indicate if the combined approach could be more useful.

According to the NMCP guidelines in Burkina Faso, a febrile child who is tested positive by mRDT should receive appropriate anti-malarial treatment. In our study, health workers tend to adhere well to this guideline and this is in line with previous observations done in the same study area [24, 40]. However, several mRDT negative children were also prescribed anti-malarials. Health workers are thought to do this in fear of missing a treatable cause of fever, in this case, malaria. Moreover, antibiotics are also widely prescribed to mRDT positive children in the present study. It is not known why health workers use this practice and a targeted social science study towards (changing) this behavior should be conducted to answer this question.

Although, in the present study, additional CRP testing did not provide a perfect solution to reduce over-prescription of antimicrobials. However, there is sufficient evidence that point of care CRP tests can contribute to control or reduce the unnecessary use of antibiotics and decreases the spread of antimicrobial resistance due to their ability to discriminate bacterial from non-bacterial infections in febrile patients [14, 41]. In some settings, where CRP testing is routinely performed in patients with respiratory tract infections, its use has led to a significant reduction of antibiotic prescriptions [42–45]. Furthermore, CRP testing can be cost-effective, it provides results within a reasonable period of time and it can be implemented in resource-limited settings [18, 46–48]. But its use as a single test is not advised in particular in settings where malaria is also highly prevalent [49]. Possibly a combination of mRDT with other acute-phase protein tests such as procalcitonin and/or white blood cell counts next to CRP testings, and supported by a thorough assessment of clinical signs and symptoms could further improve the management of febrile diseases and the prescription of antimicrobials in resource limited-settings [2, 14, 50, 51].

## Conclusion

In high malaria-endemic areas, the combined testing of febrile cases with mRDT-*Pf*HRP2 and CRP RDT is only useful in case both tests are negative and a malaria infection can be excluded. This approach could result in a reduction in antibiotic prescriptions in the group of febrile cases, although there is a risk of not treating children with an actual bacterial infection.

## Data Availability

The datasets and materials of our study are available from the corresponding author on reasonable request.

## Abbreviations

bBSI: Bacterial bloodstream infection
bGE: Bacterial gastro-enteritis
bUTI: Bacterial urinary tract infection
CRF: Case record form
CRP: C-reactive protein
EPEC: Enteropathogenic *Escherichia coli*
NMCP: National malaria control program
*Pf*-HRP-2: *Plasmodium falciparum* Specific histidine-rich protein 2
pGE: Parasitic gastro-enteritis
RDT: Rapid diagnostic test
vGE: Viral gastro-enteritis
